# Complete Genome Sequence of Campylobacter fetus Isolated from a Sheep

**DOI:** 10.1128/MRA.01008-20

**Published:** 2020-11-05

**Authors:** Daniela Costa, Virginia Aráoz, Maila Barcellos, Rubén Darío Caffarena, Martín Fraga, Federico Giannitti, Cecilia Monesiglio, Ruben Pérez, Caroline da Silva Silveira, Lucía Calleros

**Affiliations:** a Sección Genética Evolutiva, Instituto de Biología, Facultad de Ciencias, Universidad de la República, Montevideo, Uruguay; b Laboratorio de Genómica Microbiana, Institut Pasteur de Montevideo, Montevideo, Uruguay; c Plataforma de Investigación en Salud Animal, Instituto Nacional de Investigación Agropecuaria Estación Experimental La Estanzuela, Colonia, Uruguay; Indiana University, Bloomington

## Abstract

Campylobacter fetus is an important reproductive pathogen of ruminants that occasionally infects humans. Here, we describe the complete circularized genome of a strain of Campylobacter fetus subsp. *fetus* isolated from a sheep. The final assembly consisted of a unique contig with a length of 1,849,237 bp.

## ANNOUNCEMENT

*Campylobacter* species are *Epsilonproteobacteria* adapted to vertebrate hosts. Many of them cause disease in a wide range of livestock species and have extensive reservoirs in wildlife. Campylobacter fetus is an important animal pathogen and an opportunistic human pathogen. It produces considerable economic losses as a major reproductive pathogen of cattle and sheep. This species is currently divided into C. fetus subsp. *fetus*, C. fetus subsp. *venerealis* (1), and C. fetus subsp. *testudinum* (2). C. fetus subsp. *fetus* causes abortion mainly in sheep (3) and, to a lesser extent, cattle.

Here, we describe the closed whole genome of a strain of C. fetus subsp. *fetus* isolated from a sheep, representing an important resource for evolutionary and epidemiological studies of the species.

Fresh placenta was received at the Instituto Nacional de Investigación Agropecuaria (INIA) Animal Health Platform (Uruguay) for bacteriological analyses. The sample was spiked into Skirrow agar and incubated in a microaerobic atmosphere for 48 h at 37°C using CampyGen (Oxoid) (4). Biochemical testing was performed, including catalase, oxidase, and hydrogen sulfide production (triple-sugar iron medium), growth in the presence of 3.5% sodium chloride or 1% glycine, and growth at 25 and 42°C in Skirrow agar (4). Results were consistent with those of Campylobacter fetus subsp. *fetus*.

Bacterial colonies from five petri dishes of pure culture were suspended in 500 μl of phosphate-buffered saline solution (pH 7.4), and DNA was extracted using the QIAamp DNA minikit (Qiagen, Inc., Valencia, CA). Whole-genome sequencing (WGS) was performed with Illumina and Pacific Biosciences (PacBio) sequencing technologies. An Illumina library was prepared with the Nextera XT library prep kit without modifications. Illumina reads were obtained using a MiSeq platform at Institut Pasteur de Montevideo, obtaining 1,443,064 2 × 150-bp paired-end (PE) reads. All bioinformatic analysis software was set to default values. Read quality was assessed using FastQC v0.11.7 (5). Bases at the ends of the reads with a quality score lower than 20 and reads shorter than 50 bases were trimmed using Trimmomatic v0.39 (TRAILING:20 MINLEN:50) (6). A total of 1,421,381 PE reads remained after filtration (mean length, 145 bases) (Table 1).

**TABLE 1:** Short- and long-read preprocessing summary

Sequencing platform	Metric	Prefilter	Postfilter
Illumina MiSeq	No. of reads	1,443,064	1,421,381
	Read length range (bp)	30–151	50–151
	Read Q-score range	21–36	22–36
PacBio RS II	No. of polymerase read bases	1,608,855,393	1,563,018,757
	No. of polymerase reads	150,292	101,447
	Polymerase read *N*_50_ (bp)	21,336	21,464
	Polymerase read length (bp)	10,704	15,407
	Polymerase read quality	0.583	0.844

Long reads were obtained using PacBio RS II single-molecule real-time (SMRT) technology with P6-C4 chemistry (Pacific Biosciences) at Macrogen, Inc. (Seoul, South Korea). A library was prepared with the 20-kb SMRTbell template library kit without modifications. DNA was sheared, and 20-kb fragments were selected. Preprocessing was done using the SMRT Portal (smrtanalysis_2.3.0.140936.p5.167094) RS Subreads 1 protocol. It obtained 1,608,855,393 bases from 150,292 reads, with an average read length of 10,704 bp and an *N*_50_ value of 16,155 bp. After filtering, 97% of these data remained (Table 1).

A hybrid assembly approach was performed with Unicycler v0.4.7 assembler (7), using *bold* mode. The quality of the assembly was addressed using Quast v4.3 (8).

Assembly resulted in a unique 1,849,237-bp contig with an average G+C content of 33.3%, as expected for C. fetus subsp. *fetus* genomes.

The sequences were annotated with NCBI Prokaryotic Genome Annotation Pipeline (PGAP v4.13) (9). The genome contains 1,884 coding sequences: 1,811 hypothetical proteins, 44 tRNA genes, 3 rRNA operons as in all other *Campylobacter* species, and 2 CRISPR arrays.

### Data availability.

These whole-genome sequencing, assembly, and raw data have been deposited in DDBJ/ENA/GenBank under the BioProject number PRJNA554155.
